# Radiation-induced changes in microcirculation and interstitial fluid pressure affecting the delivery of macromolecules and nanotherapeutics to tumors

**DOI:** 10.3389/fonc.2012.00165

**Published:** 2012-11-15

**Authors:** Gabriele Multhoff, Peter Vaupel

**Affiliations:** ^1^Department of Radiotherapy and Radiooncology, Klinikum rechts der Isar, Technical University of MunichMunich, Germany; ^2^Helmholtz Zentrum München (HMGU), CCG - Innate Immunity in Tumor BiologyMunich, Germany

**Keywords:** irradiation, tumor microcirculation, transport barriers, tumor interstitial fluid pressure, macromolecular agents, intratumor pharmacokinetics

## Abstract

The immature, chaotic microvasculature of most solid tumors can present a significant impediment to blood-borne delivery, uneven distribution, and compromised penetration of macromolecular anticancer drugs and diagnostic agents from tumor microvessels across the interstitial space to cancer cells. To reach viable tumor cells in relevant concentrations, macromolecular agents are confronted with several barriers to vascular, transvascular, and interstitial transport. Amongst those (1) heterogeneous and poor blood supply, (2) distinctly reduced or even abolished hydrostatic and oncotic pressure gradients across the microvessel wall abrogating the convective transport from the vessel lumen into the interstitial space (impairment of transvascular transport), and (3) impediment of convective transport within the interstitial compartment due to elevated interstitial fluid pressure (IFP) (resulting from hyperpermeable blood vessels coupled with non-functional lymphatics) and a dense structure of the interstitial matrix are the major mechanisms hindering drug delivery. Upon irradiation, changes in these barrier functions are inconclusive so far. Alterations in vascular transport properties following fractionated radiation up to 40 Gy are quite inconsistent in terms of direction, extent, and time course. Total doses above 45 Gy can damage tumor microvessels, additionally impeding vascular delivery. Vascular permeability for macromolecules might be enhanced up to a total dose of 45 Gy. However, this effect is counteracted/abolished by the elevated IFP in solid tumors. When assessing IFP during fractionated radiotherapy in patient tumors, inconsistent alterations have been observed, both in direction and extent. From these data it is concluded that modulations in vascular, transvascular, and interstitial transport by irradiation of solid tumors are rather unclear so far. Translation of experimental data into the clinical setting thus needs to be undertaken with especial care.

## Introduction

The chaotic microvasculature of solid tumors leads to significant impediment of delivery, uneven distribution, and compromised penetration of macromolecules and nanotherapeutics from tumor microvessels across the interstitial compartment to cancer cells, especially to cells distant from microvessels. To reach viable tumor cells in relevant concentrations, diagnostic, and therapeutic agents are confronted with several obstacles: disturbed convective transport within the chaotic vascular compartment (*vascular transport*), spatio-temporally uneven distribution within the tissue, and significant shunt flow bypassing the exchange processes between the vascular bed and the extravascular space. Extravasation (*transvascular transport*) and extravascular convection (*interstitial transport*) of macromolecules and nanoparticles are mainly impaired by high interstitial fluid pressure (IFP). Furthermore, marked gradients in concentrations of macromolecules and nanoparticles exist within the extravascular space limiting anticancer therapies with increasing distance from tumor blood vessels (Jain, 1987, 1990; Vaupel, 2009b; Jain and Stylianopoulos, 2010; Vaupel and Multhoff, 2012).

**Figure 1:**
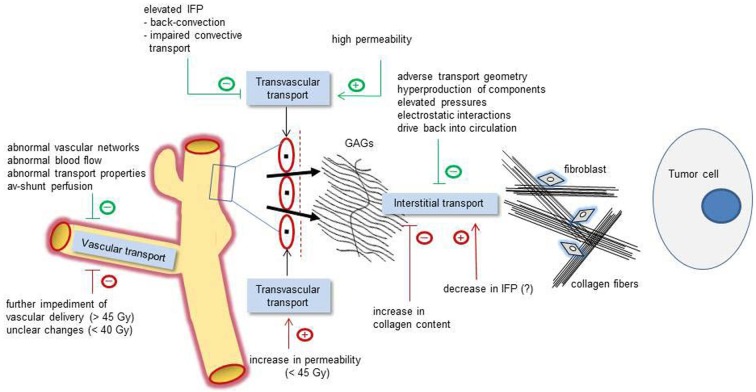
**Schematic representation of relevant pathophysiological mechanisms affecting the vascular (left), transvascular (center), and interstitial transport (right) of macromolecular compounds (e.g., anti-tumor and diagnostic large-size molecules).** Green tags: basic pathophysiological obstacles (negative signs) or facilitating mechanisms (positive signs). Red tags: irradiation-induced modulations affecting the transport properties in a positive or negative direction. Interstitial transport of macromolecules is hindered by an adverse transport geometry (including enlarged interstitial volumes and transport distances), by a hyperproduction of interstitial components (e.g., stromal cells, collagen fibers, interstitial matrix), by elevated pressures (IFP, OP, and accumulated solid stress), electrostatic interactions, and drive back into the circulation. IFP, interstitial fluid pressure; OP, oncotic pressure; GAGs, negatively charged glycosaminoglycans.

Amongst the key pathophysiological abnormalities in solid tumors related to drug transport, chaotic vascular networks, abnormal blood flow, and elevated IFP (interstitial hypertension) seem to play the dominant roles (see Figure 1). Accumulated solid stress from the growing tumor (through unlimited proliferation of cancer cells and excessive production of collagen and hyaluran), a dense interstitial structure, and contractions of the interstitial matrix mediated by stromal fibroblasts add to the transport barrier to anticancer agents (Heldin et al., 2004; Chauhan et al., 2011; Wiig and Swartz, 2012).

While some data suggest that interstitial hypertension might not be a significant barrier to therapy as has generally been proposed (Wiig and Swartz, 2012), in the following sections the impact of irradiation on the key pathophysiological characteristics mentioned above will be discussed with regard to their effect on the delivery of macromolecules and nanotherapeutics to primary and metastatic tumors.

## Vascular transport

Vascular transport, i.e., the delivery of anticancer and diagnostic agents via the blood stream, includes the convective transport to the tumor and the subsequent distribution within the tumor (“blood-borne delivery,” Vaupel and Multhoff, 2012). The development of a disorganized microvasculature and significant arterio-venous shunt perfusion leads to an inefficient delivery of (macromolecular) agents and nutrients (e.g., oxygen, glucose) through the vascular system of the tumor (see Table 1). The situation is further aggravated by flow-dependent spatio-temporal heterogeneities in the distribution of plasma-borne agents (and their metabolites). These “4D-heterogeneities” are not static, but instead are quite dynamic, and therefore more complex than has been previously assumed (for reviews see Vaupel et al., 1989; Vaupel, 2006, 2009a,b, 2012).

**Table 1:** **Obstacles in blood-borne delivery of macromolecular anticancer and diagnostic agents and modulations following irradiation (selection; Vaupel, 2006, 2009a)**.

**A. ABNORMAL VASCULAR NETWORK (“MORPHOLOGICAL ABNORMALITIES”)**
Development of an immature, disorganized microvasculature
Spatial heterogeneities
Existence of avascular spaces
Enlarged intervessel distances
Blind vessel endings
Arterio-venous anastomoses
Convoluted, elongated, and dilated microvessels
Leaky microvessels
**B. ABNORMAL BLOOD FLOW (“FUNCTIONAL ABNORMALITIES”)**
Excessive spatial and temporal heterogeneity in flow (“4D-heterogeneity”)
Slowing of blood flow, flow stops
Poor, inadequate perfusion
Sluggish perfusion
Unstable flow velocities
Arterio-venous shunt perfusion
Flow reversals
Elevated geometric and viscous resistance to flow
**C. IRRADIATION-INDUCED MODULATIONS OF BLOOD-BORNE DELIVERY**
Changes in vascular transport properties following fractionated irradiation up to 40 Gy are rather unclear
Total doses above 45 Gy may damage tumor microvessels further impeding vascular delivery

The status of the tumor microvasculature and blood flow (direction, extent, and time course of changes) upon irradiation remains largely unclear, both for single large doses (12–50 Gy) and fractionated radiation (25 fractions, 5 weeks, up to a total dose of 75 Gy), but also appears to depend on the tumor type studied, the radiation dose, the time interval between exposures, and irradiation stage (during vs. post). The literature provides quite conflicting data on whether or not radiation-related biologically or clinically relevant changes in microvascular structures and functions occur.

Descriptive and morphometric studies performed between 1927 and 1977 using experimental tumors suggested that fractionated doses commonly led to an increase in vascular density, while single large doses often destroyed the vasculature and shut down blood flow (for details see Narayan and Cliff, 1982; Fajardo and Berthrong, 1988; Baker and Krochak, 1989; Dewhirst, 1991). However, experiments using single large dose irradiation are quite inconclusive since changes in tumor blood flow were both dose-and time-dependent (Vaupel et al., 1984). In a recent review, a very contradictory data set for single large dose local irradiation in the experimental setting has been presented (Kozin et al., 2012).

In conventional fractionation schedules, tumor microvessels are distinctly damaged above doses of 40–45 Gy (Zywietz et al., 1994). Above this “critical cumulated dose” tumor oxygenation and ATP levels progressively decreased (Thews et al., 1999), clearly showing that these parameters are critically determined by the efficacy of tumor blood flow. Continuous hyperfractionation (2 daily fractions of 2.5 Gy, up to 60 Gy), however, induced only relatively discreet alterations of the tumor microvasculature (Lorke et al., 1999).

Published data on changes in tumor blood flow and oxygenation upon radiation therapy in the clinical setting showed no clear direction in observed alterations (Feldmann et al., 2000; Molls et al., 2000). From this compilation of data, there is evidence that changes in tumor microcirculation (i.e., vascular transport properties) following γ-irradiation (fractionated doses, up to 40 Gy) are rather unclear so far due to obvious variabilities in the direction, extent, and time course of changes observed. There is at least some consensus that upon conventional fractionation with total doses above 45 Gy microvessels are damaged, further impeding vascular delivery of blood-borne anticancer (macro-) molecules.

## Transvascular transport

Therapeutic (and diagnostic) molecules and nanomedicines cross the leaky vessel walls by two major mechanisms: diffusion and convection. Large pore sizes of tumor microvessels facilitate these transport processes. Diffusion is the prevailing molecular transport modality of small-size molecules driven by concentration gradients. Convection is driven by hydrostatic pressure gradients and is the dominant mode of transport for large molecules, liposomes, and other nanoparticles (Kuszyk et al., 2001). Due to the elevated interstitial fluid pressure (IFP, interstitial hypertension, see section below), transvascular pressure gradients are approaching zero. As a result of this “equilibration” of hydraulic pressures, significant hindering of the transport of macromolecules and nanoparticles into the extravascular space by convection has to be considered (see Table 2). For this reason, the main mechanism of mass transport across vessel walls is diffusion (for a review see Vaupel and Multhoff, 2012). This process is significantly slower than convection, especially for macromolecules and nanoparticles (Jain and Stylianopoulos, 2010). Vessel wall hyperpermeability (enhanced porosity) is thus counteracted by elevated IFP in tumors (and by the large size of nanoparticles).

**Table 2:** **Obstacles to transvascular transport (extravasation) of macromolecular therapeutic and diagnostic agents in solid tumors and modulations upon irradiation (selection, Vaupel and Multhoff, 2012)**.

**A. MECHANISMS FACILITATING EXTRAVASATION**
Presence of abundant fenestrae, wide channels, and large pores in the microvascular wall
High permeability (leakiness) of microvessels (vascular permeability is at least 10 times higher than interstitial permeability; Lunt et al., 2008)
**B. MECHANISMS HINDERING EXTRAVASATION**
Leakiness of microvessels is heterogeneous
Impaired transluminal convective transport of macromolecules (due to elevated IFP, see Table 3)
Decreased transfer of large-sized, anionic, and neutral particles
Intravasation back to vascular compartment (due to elevated IFP, see Table 3), “back-convection” from the interstitial space into the circulation
**C. IRRADIATION-INDUCED MODULATION OF EXTRAVASATION**
Radiation-induced increase in vascular permeability might enhance extravasation up to a total dose of 45 Gy
However, enhanced permeability is counteracted by elevated interstitial fluid pressure (IFP)
Due to elevated IFP transluminal transport can be reversed (intravasation instead of extravasation)

Vascular permeability decreases with increasing size of the transported nanoparticles (according to the Organization for Standardization, nanomedical approaches use particles from 1 to 100 nm; e.g., gold nanoparticles 2.5 nm, monoclonal antibodies 10–15 nm, oncolytic viruses 30–40 nm, magnetic nanoparticles for drug targeting 15–100 nm, liposome-encapsulated doxorubicin 80–130 nm, gadolinium-based nanoparticles 115 nm, and albumin-paclitaxel nanoparticles 130 nm). Furthermore, permeability is higher for cationic compounds than for their anionic or neutral counterparts (Jain and Stylianopoulos, 2010).

Upon fractionated γ-irradiation, time- and dose-dependent changes in vascular permeability have been described in the experimental setting due to direct vessel wall damage and the action of indirect inflammatory stimuli (Lorke et al., 1999). A discrete increase in leakiness (associated with interstitial edema) has been observed already after a total dose of 15 Gy, with more pronounced leakiness at higher radiation doses. Upon radiation with a total dose of 30 Gy, hyperpermeability was further increased. Prolonged irradiation was eventually associated with progressive destruction of the vascular wall and disruption of the basal lamina.

In principle, radiation-triggered increases in vascular permeability may enhance extravasation of anti-cancer macromolecules up to a total dose of approximately 45 Gy. However, this facilitation is severely counteracted or totally abolished by mechanisms occurring in the interstitial compartment as outlined in the following section.

## Interstitial transport

The interstitial compartment of tumors differs significantly from that of normal tissue (Vaupel and Multhoff, 2012). As a result of (1) vessel leakiness, (2) lack of functional lymphatics, (3) interstitial fibrosis, (4) contraction of the interstitial matrix mediated by stromal fibroblasts, and (5) cell proliferation in a confined space, most solid tumors develop an elevated interstitial/hydrostatic fluid pressure (IFP), which is in contrast to normal tissues where IFP is close to atmospheric pressure (Jain, 1987, 1990; Heldin et al., 2004; Milosevic et al., 2004; Cairns et al., 2006; Wiig and Swartz, 2012).

As already mentioned above, increased IFP within solid tumors decreases extravasation. In addition, high IFP severely inhibits interstitial transport of larger molecules (e.g., antibodies, antibody drug conjugates, and liposomes) by convection (see Table 3). Macromolecules rely more heavily on convection as opposed to simple diffusional transport of low-molecular weight drugs. Compounds larger than 60 nm in diameter are not able to effectively diffuse through the extracellular matrix of highly fibrotic tumors. Interstitial transport of macromolecules is further impaired by a much denser network of interconnected collagen fibers in the extracellular matrix of tumors (as compared to normal tissues) leaving them in higher concentrations in perivascular areas only (Jain and Stylianopoulos, 2010). The transport of compounds with sizes of up to 1000 nm is further hindered by highly negatively charged heparan sulfate in the matrix.

**Table 3:** **Obstacles in interstitial transport of macromolecular anti-cancer agents and nanomedicines and modulations following irradiation (selection, Vaupel and Multhoff, 2012)**.

**A. PATHOMORPHOLOGICAL CHARACTERISTICS OF THE INTERSTITIAL COMPARTMENT**
Enlarged interstitial volume
Enlarged interstitial transport distances
Hyperplasia of stromal cells
High stromal fraction
Dense network of collagen fibers
Hyperproduction of interstitial matrix
Non-functional lymphatics in the tumor center
**B. PATHOPHYSIOLOGICAL FEATURES OF THE INTERSTITIAL COMPARTMENT**
Elevated hydrostatic fluid pressure (IFP, 5–40 mmHg in solid tumors vs. −3 to +1 mmHg in most normal tissues)
Elevated oncotic (colloid osmotic) pressures (approximately 20.5 mmHg in tumors vs. 8 mmHg in subcutis; Stohrer et al., 2000)
Equilibrium between oncotic pressures of plasma and tumor interstitium
Transmural coupling between IFP and microvascular pressure leading to slowing/stoppage and even reversals of microvascular blood flow
Convective drive of anti-cancer agents back into the circulation
High visco-elasticity caused by glycosaminoglycans, e.g., hyaluronan
Severely hampered convective transport within the interstitial compartment
(Poor) diffusion largely responsible for interstitial transport in the bulk of tumors
Diffusivity (diffusion coefficient) decreases with increasing size of macromolecules
Diffusion rate for macromolecules correlates with orientation of collagen
Electrostatic interaction of charged particles with charged compounds of the interstitium
Electrostatic binding of macromolecules/nanoparticles by heparan sulfate
Escape of macromolecules at the tumor edge into the surrounding normal tissue
Diversion of blood flow from center to periphery of tumors due to elevated IFP
**C. MODULATION OF INTERSTITIAL TRANSPORT UPON IRRADIATION**
Inconclusive results when assessing IFP during fractionated radiotherapy in patients with cancers of the uterine cervix
(decrease in IFP in four out of seven patients, increase in IFP in three patients; Roh et al., 1991)
Decrease in IFP above a threshold of 10 Gy upon single dose or fractionated radiation of human colon cancer xenografts (Znati et al., 1996)
Reduced convective and diffusive transport of macromolecules following single dose or fractionated irradiation
(reduced interstitial fluid transport, increased collagen content; Znati et al., 2003)

Heterogeneous mobility and distribution of large-sized molecules is additionally caused by two phases in the matrix: a more aqueous phase is found in regions with low fiber content (“fast” compartment with relatively high diffusivity), and a more viscous phase is due to a high concentration of collagen fibers in a dense matrix (“slow” compartment with high retention of compounds). Collagen content in tumors is much higher and collagen fibers are much thicker than in normal tissue leading to an increased mechanical stiffness of the tissue (Netti et al., 2000; Heldin et al., 2004). The interstitium also contains abundant stromal cells and enzymes that can affect the activity and delivery of agents to the tumor cells (Kuszyk et al., 2001).

It is assumed that IFP is almost uniform throughout a tumor and that relevant gradients of IFP do not exist. However, IFP drops precipitously at the tumor/normal tissue interface. For this reason, the interstitial fluid oozes out of the tumor into the surrounding normal tissue, carrying away anticancer agents, growth factors or released heat shock proteins, and cancer cells with it (Fukumura and Jain, 2007). Shedded cancer cells may mediate metastasis. As another consequence of this peripheral drop in IFP, blood flow may be diverted away from the tumor center toward the periphery where anticancer agents may be lost from larger vessels.

Transmural coupling between IFP and microvascular pressure can critically reduce perfusion pressure between up- and downstream tumor blood vessels leading to flow stasis and thus, inadequate delivery of anticancer agents, in addition to the mechanisms impairing blood flow already mentioned above.

In the experimental setting, radiocurability of human tumor xenografts decreases with increasing IFP (Rofstad et al., 2009, 2010). In these experiments, IFP showed a strong positive correlation to the extent of acute hypoxia in the tumors investigated (Rofstad et al., 2009), an increased number of clonogenic cells (Rofstad et al., 2010), stimulation of proliferation, occurring presumably via modulation of signaling pathways (Nathan et al., 2005), and upregulation of VEGF-A expression (Nathan et al., 2008).

Studies in patients with cervix cancers have explored the relationship between IFP and outcome following radiotherapy (Milosevic et al., 2001; Yeo et al., 2009). In these studies, IFP was found to be a strong, negative, and independent prognostic factor for local control and distant metastasis. Several compounds have been shown to decrease tumor IFP in patients (for a review see Heldin et al., 2004). This reduction in IFP has been attributed to a substantial decrease in vascular permeability, lowered microvascular pressure and changes in the extracellular matrix.

Assessing interstitial hypertension during fractionated radiotherapy in patients with cervix cancers showed inconclusive results, since only 4 out of 7 patients experienced a drop in IFP during treatment, whereas in 3 patients IFP distinctly increased (Roh et al., 1991). Measurements after single dose or fractionated radiation in human colon cancer xenografts yielded a reduction in IFP above a threshold of 10 Gy. Below this threshold there was no significant change in IFP (Znati et al., 1996). A decrease in microvascular pressure has been discussed as a plausible explanation for the radiation-induced reduction in IFP by these authors. Furthermore, the authors argued that this radiation-related decrease in IFP may have been responsible for an improved uptake of monoclonal antibodies following single dose or fractionated irradiation as reported earlier by others. In contrast to these data, in a later publication by this group a reduced interstitial fluid transport and increased collagen content in tumors has been communicated (Znati et al., 2003), implicating a reduced transport of macromolecular agents in tumors upon radiation.

## Concluding remarks

Preceding cellular pharmacodynamics, three important pharmacokinetic steps govern the delivery of anti-cancer drugs and diagnostic agents to tumor cells: vascular, transvascular, and interstitial transport. Barriers to delivery of macromolecular drugs mainly arise from immature, chaotic vascular networks and abnormal tumor blood flow, hyperpermeability of leaky microvessels, and elevated fluid pressure within the interstitial compartment abrogating convective transport. Upon tumor irradiation, changes in these barriers and thus in transport properties are inconsistent so far, so that definite conclusions for the clinical (and experimental) setting cannot be drawn. Therefore, transport mechanisms for (macro-) molecules should increasingly receive attention. One of the goals of translational cancer research is to obtain a better understanding of the compromised delivery and distribution of anti-cancer compounds in solid tumors (i.e., intratumor pharmacokinetics) in order to improve patients' outcomes.

### Conflict of interest statement

The authors declare that the research was conducted in the absence of any commercial or financial relationships that could be construed as a potential conflict of interest.
